# Tissue xanthine oxidoreductase activity in a mouse model of aristolochic acid nephropathy

**DOI:** 10.1002/2211-5463.13083

**Published:** 2021-02-01

**Authors:** Takeo Ishii, Tomohiro Kumagae, Hiromichi Wakui, Shingo Urate, Shohei Tanaka, Eriko Abe, Toru Suzuki, Takahiro Yamaji, Sho Kinguchi, Ryu Kobayashi, Kotaro Haruhara, Takashi Nakamura, Shuzo Kobayashi, Kouichi Tamura

**Affiliations:** ^1^ Department of Medical Science and Cardiorenal Medicine Yokohama City University Graduate School of Medicine Kanagawa Japan; ^2^ Department of Medicine Yokohama Daiichi Hospital Kanagawa Japan; ^3^ Department of General Internal Medicine Shonan Kamakura General Hospital Kanagawa Japan; ^4^ Medical Affairs Department Sanwa Kagaku Kenkusho., Co., Ltd Aichi Japan

**Keywords:** aristolochic acid, mouse model, nephropathy, xanthine oxidoreductase

## Abstract

Xanthine oxidoreductase (XOR) is a critical enzyme in purine metabolism and uric acid production, and its levels are reported to increase during stress, thereby promoting organ damage. Herein, we investigated the activity of XOR in a mouse model of aristolochic acid I (AA)‐induced nephropathy, a type of nephrotoxic chronic kidney disease (CKD). A persistent decrease in renal function was observed in mice up to 4 weeks after 4 weeks of AA (2.5 mg kg^−1^) administration. Renal histology revealed an increase in tubular interstitial fibrosis over time. Although AA administration did not change XOR activity in the plasma, heart, liver, or muscle, XOR activity was persistently increased in renal tissue. Our results suggest that the renal tissue‐specific increase in XOR activity is involved in the progression of tubulo‐interstitial disorders, specifically fibrosis.

## Graphical abstract

We investigated the activity of xanthine oxidoreductase (XOR) in a mouse model of aristolochic acid I (AA)‐induced nephropathy, a type of chronic kidney disease (CKD). After 4 weeks of AA (2.5 mg·kg^−1^) administration, renal histology revealed an increase in tubular interstitial fibrosis and XOR activity was persistently increased in renal tissue. Our data suggest that XOR activity is involved in the progression of tubulointerstitial disorders.

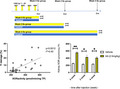

**Abbreviations**
AA: aristolochic acid
BUN: blood urea nitrogen
CKD: chronic kidney disease
Col‐1: collagen‐1
Cr: creatinine
HIF‐1α: hypoxia‐inducible factor‐1 alpha
NLRP3: NLR family pyrin domain containing 3
OAT1: organic anion transporter 1
PTC: peritubular capillary
ROS: reactive oxygen species
TG2: transglutaminase type 2
TGF‐β: transforming growth factor‐β
TP: total protein
VEGF: vascular endothelial growth factor
XDH: xanthine dehydrogenase
XO: xanthine oxidase
XOR: xanthine oxidoreductase

## Introduction

Chronic kidney disease (CKD) is prevalent worldwide, and various associated diseases including fibrotic disorders pose a challenge in kidney tissues [1]. The increase in CKD has led to an increased number of patients on dialysis, and an increased financial burden, owing to dialysis costs [2]. Therefore, there is an urgent need to reduce and prevent renal dysfunction, which leads to end‐stage renal failure. To study CKD, we focused an animal model of aristolochic acid (AA)‐induced nephropathy, which is characterized by progressive fibrosis. AA nephropathy is a problem on the Balkan Peninsula [3, 4] and in China where the Mu Tong renal disorder [5] is endemic, leading to fibrosis and irreversible end‐stage renal failure [6]. Additionally, modern lifestyle leads to hypertension, diabetes, hyperuricemia, and gout, which are posing problems globally. In addition to causing gout, hyperuricemia was recently reported as a risk factor for the development of nephrosclerosis and hypertension [7, 8, 9]. Uric acid causes the formation of crystal‐associated disease networks through the deposition of monosodium urate crystals [10], resulting from purine metabolism. This causes oxidative stress‐related tissue damage and tissue inflammation due to the generation of superoxides by xanthine oxidoreductase (XOR) [11, 12]. XOR was discovered approximately 100 years ago, and xanthine dehydrogenase (XDH) and xanthine oxidase (XO) conversion were discovered approximately 30 years ago [13]. Under physiological conditions, XO exists in milk and plays a role in physiological sterilization [14, 15]. *In vivo*, XDH preferentially reacts with NAD^+^, whereas XO cannot, but produces the superoxide anion (O_2_^−^) and hydrogen peroxide (H_2_O_2_) [16, 17, 18, 19]. It is well established that O_2_^−^ and H_2_O_2_ cause tissue damage [11]. XOR expression is upregulated by ATP degradation caused by certain pathological conditions such as ischemia, hypoxia, and oxidative stress, triggered by cellular stress. Under these pathological conditions, ATP is metabolized to hypoxanthine, xanthine, and uric acid via reactions catalyzed by XOR [12]. XOR produces reactive oxygen species (ROS) such as superoxides, hydroxyl radicals, hydrogen peroxide, and peroxynitrite [20]. XOR also induces the renin‐angiotensin system in tissues, which promotes organ damage and renal sclerosis through the activation of a positive feedback loop [12]. We previously reported that treatment with XOR inhibitors can improve the survival of patients on dialysis [21]. XOR inhibition may potentially reduce the oxidative stress associated with organ damage due to renal failure. In this study, we analyzed XOR activity in the kidneys, plasma, heart, liver, and muscle in this AA‐induced nephropathy. AA enters renal tubular epithelial cells through the OAT1 channel on the basement membrane. It stops the cell cycle and forces the cell to undergo apoptosis, causing irreversible fibrosis. AA can do this in kidneys without damage to other organs. We chose AA because it shows the fibrotic mechanism to end‐stage renal failure, functioning as a model of acute kidney injury that can progress toward chronic kidney disease. The aim of this study was to investigate the relevance of XOR activity in terms of tissue damage. The AA model is a model of acute kidney injury with the potential to progress toward chronic kidney disease. We investigated tissue damage in this model based on time‐dependent XOR activity.

## Materials and methods

### Animals

**Fig. 1:**
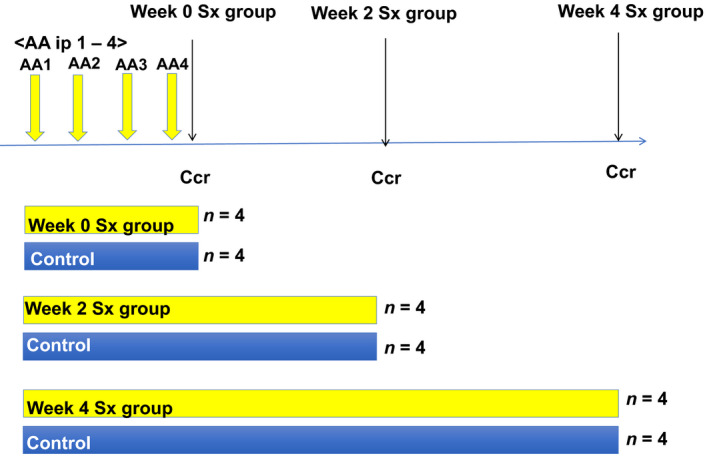
Experimental procedure and timeline for the mouse model. Male C57BL/6 mice were assigned to either an aristolochic acid I (AA) treatment group or the vehicle control group. Each group was divided into three subgroups depending on whether they were sacrificed at 0, 2, or 4 weeks after their fourth injection. AA (2.5 mg·kg^−1^) was administered intraperitoneally (ip) once a week for four consecutive weeks. For 24 h before sacrifice (Sx), urine was collected in metabolic cages as described previously. For the 0‐week AA group *n* = 4, 2‐week group *n* = 3, 4‐week group *n* = 4, and control *n* = 4. Serum creatinine clearance (Ccr) levels were measured immediately after the last AA injection.

Male 12‐week‐old C57BL/6 mice (purchased from Charles River Laboratories) were assigned to either an AA treatment group or the vehicle control group. Each group was divided into three subgroups depending on whether they were sacrificed at 0, 2, or 4 weeks after the fourth injection. AA (2.5 mg·kg^−1^) was administered via intraperitoneal injection once a week for four consecutive weeks as previously described [22]. This dosing regimen was used in order to induce chronic fibrosis with a low rate of death. XOR activity was evaluated following AA administration, and tissues were examined for XOR‐related damage. Before sacrifice (24 h), urine was collected in metabolic cages as described previously [23] (0‐week group *n* = 4, 2‐week group *n* = 3, 4‐week group *n* = 4, control *n* = 4) (Fig. 1). Mice were subjected to a 12‐h light/12‐h dark cycle at 25℃ and were provided a standard diet (0.3 % NaCl, 3.6 kcal g^−1^, 13.3 % energy as fat (Oriental MF; Oriental Yeast Co., Ltd., Andover, MA, USA)) with free access to water. This study was approved by the Yokohama City University Laboratory Animal Committee and was conducted as per the Ministry of Health, Labor, and Welfare Laboratory Animal Guidelines. Serum albumin was measured using bromocresol green (BCG); urine albumin was measured using a turbidimetric immunoassay; and serum and urine creatinine (Cr) were measured using enzymatic methods.

### Metabolic cage analysis

Metabolic cage analysis was performed as previously described (Techniplast, Paola, Malta) [23, 24]. Daily food and water intake were measured.

### Histological analysis

Histological analysis was performed as described previously [25, 26]. The kidney was fixed with 4% PFA and embedded in paraffin. Sections (4‐lm‐thick) were stained with Masson’s trichrome. For analysis of renal structures, renal fibrotic areas were measured digitally using a fluorescence microscope (BZ‐9000; Keyence, Osaka, Japan). The obtained image was red/blue‐stained, and the blue was extracted. After binarization considering the overall balance, the tubular epithelium and the blue‐stained part of the lumen were found to not be fibrotic and were trimmed and excluded in order to quantify the fibrotic area. Perivascular fibrosis was included in the quantification. Tubulo‐interstitial damage was defined as the ratio between the stained area and the area of the whole specimen. Further, we estimated simple regression between tissue XOR activity and tubulo‐interstitial damage (%).

### Real‐time quantitative reverse transcription‐polymerase chain reaction (RT‐qPCR)

Total RNA was extracted from renal tissues using ISOGEN (Nippon Gene, Tokyo, Japan), and cDNA was synthesized using the SuperScript III first‐strand synthesis system (Invitrogen). RT‐qPCR was performed using a TaqMan PCR master mix and a designed TaqMan probe (Applied Biosystems, Foster City, CA, USA) on an ABIPRISM 7000 sequence detection system. Expression levels of target mRNAs were normalized to the 18S rRNA. The TaqMan probes used for PCR were as follows: transforming growth factor‐beta 1 (TGF‐beta 1), MM01178820_m1; collagen‐1a, Mm00801666_g1; hypoxia‐inducible factor 1 alpha subunit inhibitor (HIF‐1a), Mm01198376_m1; and angiotensinogen (AGT), Mm00599662_m1; RAS‐related C3 botulinum toxin substrate 1 (RAC 1) Mm01201656_m1; NADPH Oxidase 1 (NOX 1) Mm00549170_m1; CCAAT/enhancer binding protein, alpha C/EBPα Mm00514283_s1; peroxisome proliferator activated receptor gamma (PPARγ) Mm00440940_m1; and sterol regulatory element binding transcription factor 1 (SREBF1), Mm00550338_m1.

### XOR activity measurement

Xanthine oxidoreductase activity was measured three times (0 week, 2 weeks, 4 weeks after AA administration) (*n* = 4 in each group) in the heart, liver, kidney, and muscle tissues according to a previously described method [27]. In brief, the kidney, liver, heart, or muscle homogenates, or plasma was added to a mixture of [^13^C_2,_^15^N_2_] xanthine, NAD+, and oxonate in Tris buffer (pH 8.5) and was incubated at 37℃ for 30 min. Then, methanol containing [^13^C_2_,^15^N_2]_UA was added, and the mixture was centrifuged at 3000 × *g* at 4℃ for 15 min. The amount of [^13^C_2_,^15^N_2_]UA produced in the supernatant was measured using LC/TQMS (Nexera/QTRAP4500, SHIMADZU/SCIEX). XOR activity was expressed as [^13^C_2_,^15^N_2_]UA nmol/min/mg protein [27].

### Statistical analysis

The results of the AA and control groups were compared using an unpaired *t*‐test. *P*‐values < 0.05 were considered statistically significant. All analyses were performed using Prism 8 Ver. 8.3.1 (Yokohama, Japan).

## Results

**Fig. 2:**
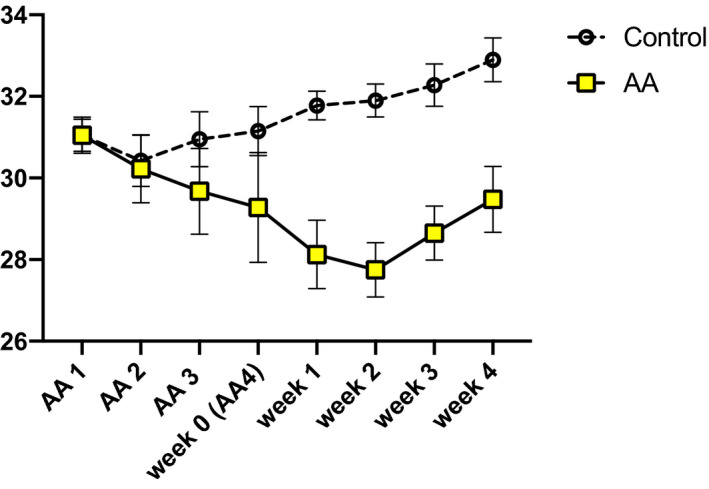
Bodyweight changes during the observation period. The mice were followed up 4 weeks after the final AA injection. The bodyweights in the vehicle and aristolochic acid I (AA) groups were 31.1 ± 0.4 g and 31.1 g ± 0.4, respectively, at the first injection. By week 4, the control group bodyweights were 31.2 ± 0.6 g, but the AA group bodyweights were 29.3 ± 1.4 g. Two weeks later (during the observation period), the bodyweights were 31.9 ± 0.4 g in the vehicle group, whereas those in the AA group reached a minimum of 27.8 ± 0.7 g (*P* < 0.05). 0‐week group *n* = 4, 2‐week group *n* = 3, 4‐week group *n* = 4, control *n* = 4. Error bars represent SEM.

**Fig. 3:**
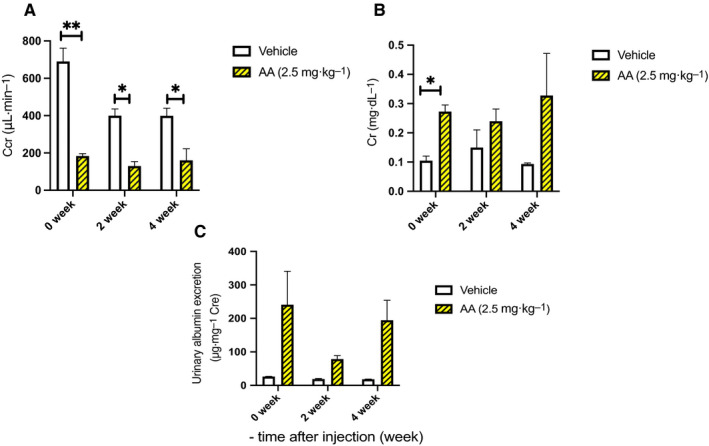
Changes in serum creatinine (Cr), Cr clearance (Ccr), and urinary albumin excretion. (A) Serum Cr levels were measured immediately after the last aristolochic acid I (AA) injection. The Cr level was 0.11 ± 0.02 mg·dL^−1^ in the vehicle group and was significantly higher in the AA group (0.30 ± 0.02 mg·dL^−1^; *P* < 0.05). The levels continued to increase in the experimental group, at 2 weeks and 4 weeks after the end of AA administration. (B) First Ccr, which was measured at the end of the AA administration, was 690.1 ± 71.4 µL·min^−1^ in the vehicle group and 183.8 ± 12.9 µL·min^−1^ in the AA groups. The levels in the AA groups remained low at 2 weeks and 4 weeks after AA administration (*P* < 0.05; Table 1 and Fig. 3a, 3b). (C) Urinary albumin excretion was measured immediately after four AA injections. The values were 26.0 ± 0.9 and 240.8 ± 100.0 µg·mg^−1^ Cr in the vehicle and AA groups, respectively. Two weeks later, the urinary albumin excretion was 18.8 ± 1.7 µg·mg^−1^ and 78.3 ± 10.6 µg·mg^−1^ Cr in the vehicle and AA groups, respectively (*P* < 0.05). At 4 weeks, the urinary albumin excretion was 18.3 ± 0.6 µg·mg^−1^ and 194.3 ± 59.8 µg·mg^−1^ Cr in the vehicle and AA groups, respectively (*P* < 0.05; Fig. 3C). 0‐week group *n* = 4, 2‐week group *n* = 3, 4‐week group *n* = 4, control *n* = 4. Error bars represent SEM. Statistical analysis was performed using multiple *t*‐test in each week. *Represents *P* < 0.05, and ** represents *P* < 0.001.

For the mice sacrificed at 4 weeks after their fourth injection, the bodyweights in the vehicle and AA groups were 31.1 g ± 0.4 g and 31.1 g ± 0.4 g, respectively, at the first injection. Two weeks later (after the fourth injection and during the observation period), the bodyweight was 31.9 g ± 0.4 g in the vehicle group, whereas it reached a minimum of 27.8 g ± 0.7 g (*P* < 0.05) in the AA group (Fig. 2). Serum Cr levels were measured immediately after the last AA injection. The Cr level was 0.11 g ± 0.02 mg·dL^−1^ in the vehicle group and was significantly higher in the AA groups (*P* < 0.05). The levels continued to increase in the experimental group at 2 and 4 weeks after the end of AA administration. After the final AA administration, serum blood urea nitrogen (BUN) was 25.7 mg·dL^−1^ ± 0.2 mg·dL^−1^ and 53.8 mg·dL^−1^ ± 5.1 mg·dL^−1^ in the vehicle and AA groups, respectively (*P* < 0.05). BUN continued to increase in the AA group during the follow‐up observation period. First Cr clearance (Ccr), which was measured at the end of the AA administration, was 690.1 µL·min^−1^ ± 71.4 µL·min^−1^ in the vehicle group and 183.8 µL·min^−1^ ± 12.9 µL·min^−1^ in the AA groups. The Ccr levels in AA groups remained low at 2 weeks and 4 weeks after AA administration (*P* < 0.05; Table 1 and Fig. 3A,B).

**Table 1:** Activities of xanthine oxidoreductase (XOR) in the aristolochic acid (AA) nephropathy model

Variable	(Time of sacrifice)	Vehicle			AA		
		0 week	2 weeks	4 weeks	0 week	2 weeks	4 weeks
Survival rate, %		100	100	100	100	75	100.0
Body weight, g		27.1 ± 0.2	31.5 ± 0.3	31.1 ± 0.4	28.8 ± 0.6	28.6 ± 0.8	31.1 ± 0.4
Body weight change rate (%)		‐2.0 ± 1.7	1.1 ± 1.4	6.0 ± 1.5	‐10.1 ± 1.8	‐5.9 ± 2.7	‐5.1 ± 1.9
Urine volume (μL·min^−1^)		2.0 ± 0.2	1.2 ± 0.3	0.7 ± 0.1	2.3 ± 0.1	1.1 ± 0.5	0.9 ± 0.1
Serum creatinine (mg·dL^−1^)		0.1 ± 0.0	0.2 ± 0.1	0.1 ± 0.0	0.3 ± 0.0*	0.2 ± 0.1	0.3 ± 0.1
Serum blood urea nitrogen (mg·dL^−1^)		25.7 ± 0.2	44.5 ± 13.1	22.5 ± 1.3	53.8 ± 5.1	48.8 ± 12.4	37.1 ± 8.6
Creatinine clearance (mL·min^−1^)		690.1 ± 71.4	399.3 ± 36.3	399.1 ± 40.5	183.8 ± 12.9**	129.4 ± 24.5*	160.6 ± 61.8*
Urine osmotic pressure (mOsm/KgH_2_O)		1995.3 ± 240.1	3376.3 ± 303.2	3955.3 ± 427.4	1602.0 ± 14.7	2651.0 ± 485.9	2934.8 ± 254.6
Plasma XOR activity (pmol min^−1^ mg TP^−1^)		73.3 ± 8.0	83.3 ± 2.7	68.3 ± 3.0	77.8 ± 4.2	60.7 ± 0.7	64.5 ± 2.6
Heart XOR activity (pmol min^−1^ mg TP^−1^)		238.8 ± 12.1	222.5 ± 18.8	213.5 ± 2.6	282.8 ± 23.5	242.3 ± 17.3	235.0 ± 2.3
Liver XOR Activity (pmol min^−1^ mg TP^−1^)		736.8 ± 38.1	666.5 ± 31.3	713.5 ± 38.5	911.5 ± 12.1	751.0 ± 41.7	760.0 ± 47.5
Muscle XOR activity (pmol min^−1^ mg TP^−1^)		112.8 ± 26.0	90.5 ± 10.0	110.0 ± 12.4	117.0 ± 15.3	90.3 ± 6.0	119.3 ± 8.5
Kidney XOR activity (pmol min^−1^ mg TP^−1^)		254.3 ± 10.0	231.5 ± 20.4	237.5 ± 9.9	556.8 ± 26.5*	430.0 ± 55.6	410.5 ± 30.5*

Values are expressed as the mean ± SE (*n* = 4)

* *P* < 0.05, **P < 0.001.

### Changes in the urinary albumin excretion

The urinary albumin excretion was measured immediately after four AA injections. The values were 26.0 ± 0.9 µg·mg^−1^ and 240.8 ± 100.0 µg·mg^−1^ Cr in the vehicle and AA groups, respectively. At 4 weeks, the urinary albumin excretion was 18.3 ± 0.6 µg·mg^−1^ and 194.3 ± 59.8 µg·mg^−1^ Cr in the vehicle and AA groups, respectively (*P* < 0.05; Fig. 3c).

### XOR activity in plasma and tissues

**Fig. 4:**
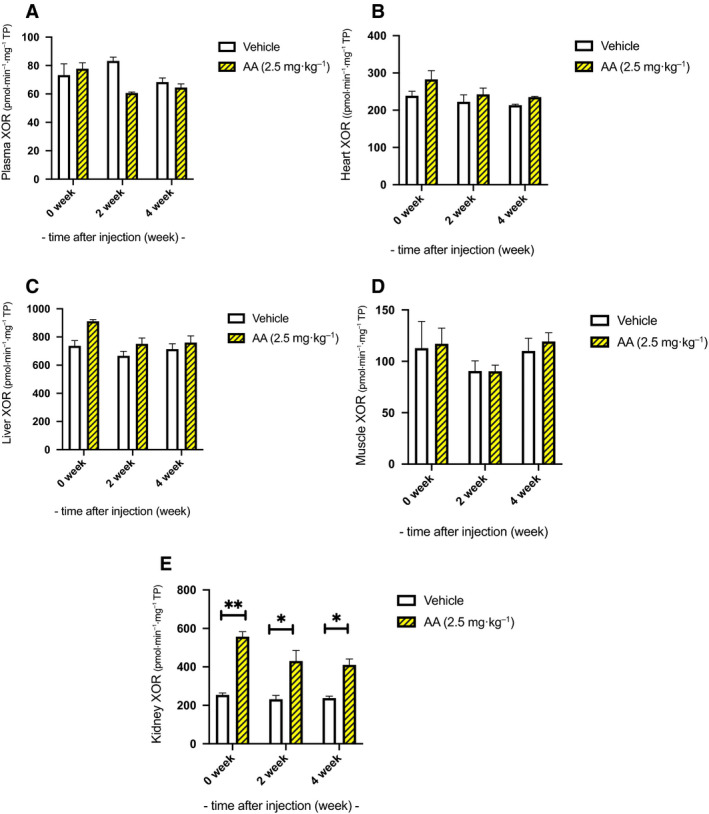
Xanthine oxidoreductase activity in plasma and tissues. (A) The plasma xanthine oxidoreductase (XOR) activity was 73.3 ± 8.0 pmol·min^−1^·mg^−1^ and 77.8 ± 4.2 pmol·min^−1^·mg^−1^ in the vehicle and aristolochic acid I (AA) groups, respectively, at the end of AA administration. No significant difference was observed in the plasma XOR activities between the two groups at any time point. (B–D) Except for the kidney, XOR activities in the examined tissues did not differ between the groups. The AA groups did not show any differences in XOR activity in the heart, liver, and muscle compared to the control group at any time point. (E) However, in renal tissue, the XOR activities in the AA groups were elevated during the observation period. At week 0, the XOR activities were 254.3 ± 19.9 pmol·min^−1^·mg^−1^ and 556.8 ± 52.9 pmol·min^−1^·mg^−1^ TP in the vehicle and AA groups, respectively (*P* < 0.0001). Two weeks later, XOR activity was 231.5 ± 20.4 pmol·min^−1^·mg^−1^ and 430 ± 55.6 pmol·min^−1^·mg^−1^ TP in the vehicle and AA groups, respectively (*P* < 0.05). At week 4, the activity was 237.5 ± 9.9 pmol·min^−1^·mg^−1^ and 410.5 ± 30.5 pmol·min^−1^·mg^−1^ TP in the vehicle and AA groups, respectively (*P* < 0.05). 0‐week group *n* = 4, 2‐week group *n* = 3, 4‐week group *n* = 4, control *n* = 4. Error bars represent SEM. Statistical analysis was performed using multiple t‐test in each week. *Represents *P* < 0.05, and **Represents *P* < 0.001.

The plasma XOR activity was 73.3 ± 8.0 pmol·min^−1^·mg^−1^ and 77.8 ± 4.2 pmol·min^−1^·mg^−1^ in the vehicle and AA groups, respectively, at the end of AA administration. No significant difference was observed in plasma XOR activities between the two groups at any time point (Fig. 4A).

Except for the kidney, XOR activities in the examined tissues did not differ between the groups. The AA groups did not show any differences in XOR activity in the heart, liver, and muscle compared to the control group at any time point (Fig. 4B–D). However, in renal tissue, the XOR activities in the AA groups were elevated during the observation period. At week 0, XOR activities were 254.3 ± 19.9 pmol·min^−1^·mg^−1^ and 556.8 ± 52.9 pmol·min^−1^·mg^−1^ TP in the vehicle and AA groups, respectively (*P* < 0.0001). Two weeks later, the XOR activity was 231.5 ± 20.4 pmol·min^−1^·mg^−1^ and 430 ± 55.6 pmol·min^−1^·mg^−1^ TP in the vehicle and AA groups, respectively (*P* < 0.05). At week 4, the activities were 237.5 ± 9.9 pmol·min^−1^·mg^−1^ and 410.5 ± 30.5 pmol·min^−1^·mg^−1^ TP in the vehicle and AA groups, respectively (*P* < 0.05; Fig. 4e).

### Expression of fibrosis markers in the renal tissue

**Fig. 5:**
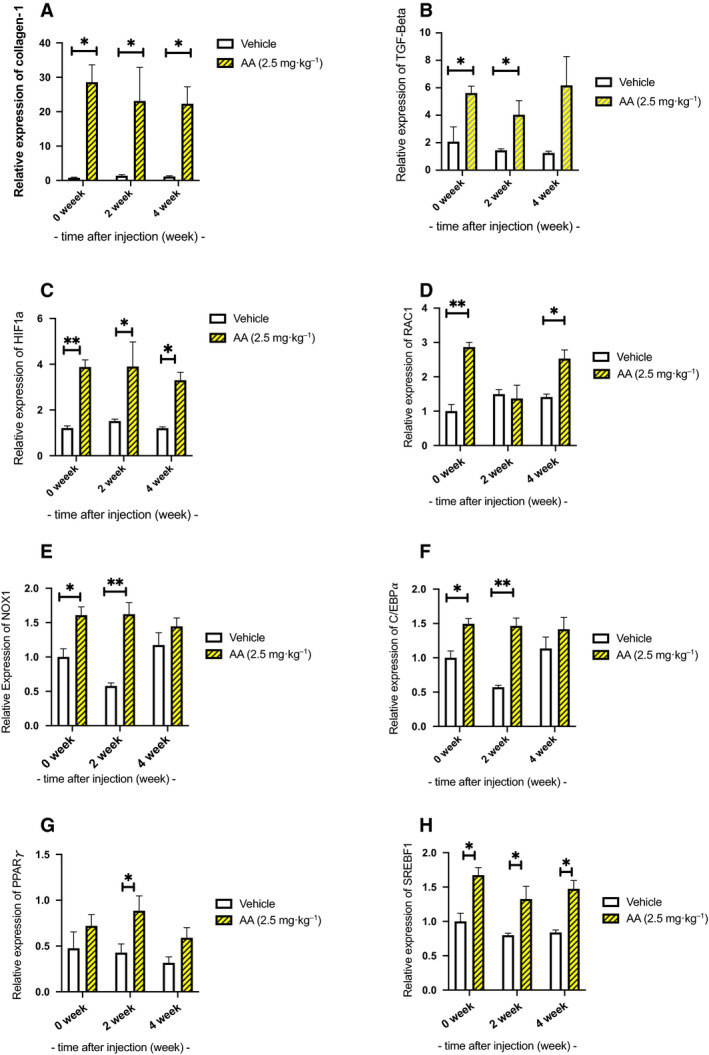
Gene expression in the renal tissue. (A) The mRNA levels of renal fibrosis‐related genes were examined. Renal expression of collagen‐1 (Col‐1) was elevated in the aristolochic acid I (AA) groups compared to the vehicle group at all time points (*P* < 0.05). (B) Additionally, renal expression of transforming growth factor‐β (TGF‐β) was increased in the AA groups compared to the vehicle group at the end of AA administration and 2 weeks later (*P* < 0.05), and showed a tendency to increase after 4 weeks. (C) The mRNA levels of hypoxia‐inducible factor‐1 alpha (HIF‐1α) were examined. Renal expression of HIF‐1α was significantly higher in the AA groups compared with the vehicle group (*P* < 0.001) at the end of AA administration, at 2 weeks, and 4 weeks (*P* < 0.05). (D, E) The mRNA levels of NADPH components were examined. Renal expression of (RAC1) and (NOX1) was elevated in the aristolochic acid I (AA) groups compared to the vehicle group (*P* < 0.05). (F–H) The mRNA levels of adipogenesis and lipogenesis markers were examined. Renal expression of (C/EBPα), (PPARγ), and (SREBF1) was elevated in the aristolochic acid I (AA) groups compared to the vehicle group (*P* < 0.05). 0‐week group *n* = 4, 2‐week group *n* = 3, 4‐week group *n* = 4, control *n* = 4. Error bars represent SEM. Statistical analysis was performed using multiple *t*‐test in each week. * represents p < 0.05, and **Represents *P* < 0.001.

The mRNA expression levels of renal fibrosis‐related genes were examined. Renal expression of collagen‐1 (Col‐1) was elevated in the AA groups compared to that in the vehicle group at all time points (*P* < 0.05; Fig. 5A). Additionally, the renal expression of transforming growth factor‐β (TGF‐β) was increased in the AA groups compared to that in the vehicle group at the end of AA administration and after 2 weeks (*P* < 0.05), and showed a tendency to increase after 4 weeks (Fig. 5B).

### Expression of hypoxia‐inducible factor (HIF‐1α) in the renal tissue

The mRNA expression levels of HIF‐1α were also examined. Renal expression of HIF‐1α was significantly higher in the AA groups compared with that in the vehicle group (*P* < 0.001) at the end of AA administration, at 2 weeks and 4 weeks (*P* < 0.05; Fig. 5C).

### Expression of NADPH components in the renal tissue

*RAC1* and *NOX1* gene expression in the AA group were elevated continuously compared with the vehicle group (*P* < 0.05; Fig. 5D,E).

### Expression of adipogenesis markers in the renal tissue

Adipogenesis markers C/EBPα and PPARγ gene expression, and lipogenesis marker SREBF1 gene expression in the AA group were elevated continuously compared with the vehicle group (*P* < 0.05; Fig. 5F, G, H).

### Renal tissue fibrosis

**Fig. 6:**
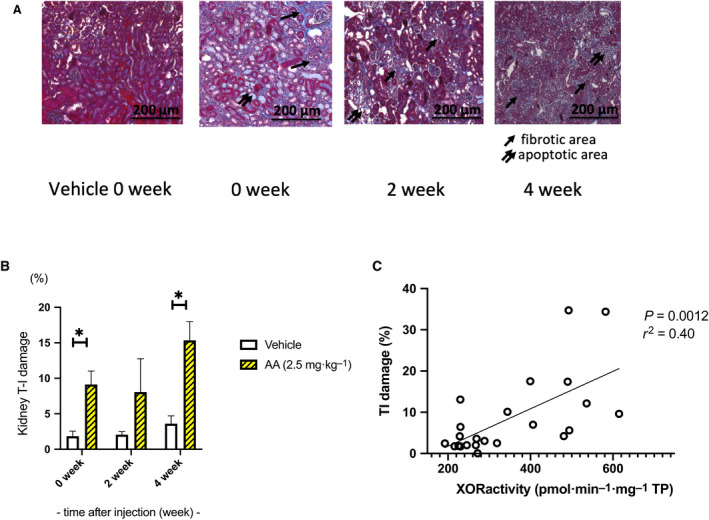
Renal tissue fibrosis. (A, B) The interstitial renal fibrosis areas were 1.8 ± 0.7% and 9.1 ± 1.9% (*P* < 0.05) in the vehicle and AA groups, respectively, at the end of AA administration. Two weeks later, the areas were 2.1 ± 0.4% and 8.1 ± 4.7% in the vehicle and AA groups, respectively. At 4 weeks, the interstitial renal fibrosis areas were 3.6 ± 1.1 and 15.3 ± 2.6% (*P* < 0.05) in the vehicle and AA groups, respectively. Scale bar indicated 200 μm. (c) Kidney tissue XOR activity was significantly correlated with interstitial fibrotic area (%) using microscopic evaluation of Masson trichrome stain (*P* = 0.0012 *r* ^2^ = 0.40). This suggests that XOR activity contributed to tissue damage leading to end‐stage renal disease. 0‐week group *n* = 4, 2‐week group *n* = 3, 4‐week group *n* = 4, control *n* = 4. Error bars represent SEM. Statistical analysis was performed using multiple t‐test in each week. *represents *P* < 0.05, and ** represents *P* < 0.001.

The interstitial renal fibrosis areas were 1.8 ± 0.7% and 9.1 ± 1.9% (*P* < 0.05) in the vehicle and AA groups, respectively, at the end of AA administration. A wide range of tubular epithelial cells was lost via apoptosis. Two weeks later, the fibrotic areas were 2.1 ± 0.4% and 8.1 ± 4.7% in the vehicle and AA groups, respectively. At 4 weeks, the interstitial renal fibrosis areas were 3.6 ± 1.1% and 15.3 ± 2.6% (*P* < 0.05) in the vehicle and AA groups, respectively. The tubular apoptotic area at 0 weeks was replaced with the fibrotic area via tissue remodeling (Fig. 6A,B). Microscopic evaluation of Masson trichrome stain showed that kidney tissue XOR activity was significantly correlated with the interstitial fibrotic area (%) (*P* < 0.0012, *r* ^2^ = 0.40) (Fig. 6C).

## Discussion

The results of the present study showed that renal XOR activity was enhanced, concomitant with the development of renal dysfunction and fibrosis in an AA nephropathy mouse model. Intriguingly, renal XOR activity was sustainably enhanced even after AA injection, which may be involved in the formation of long‐term renal fibrosis in AA nephropathy. AA is incorporated into tubular epithelial cells via the organic anion transporter 1 (OAT1) channel expressed on the renal tubular basement membrane [28, 29]. After AA administration, cellular cyclin B1 levels increase, reflective of the cell cycle arrest of tubular epithelial cells before mitosis, and apoptosis occurs intermittently [30]. This mechanism is the reason why this AA nephropathy model is kidney‐specific and useful for investigating the mechanism of renal fibrosis. We have not tested mice younger than 12 weeks; nevertheless, these mice would likely have exhibited similar patterns of renal tubular epithelial cell apoptosis and basement membrane drop. However, it is not known whether epithelial cell turnover is activated more rapidly in younger mice. Tubular epithelial cell apoptosis results in cell cycle arrest of the peritubular capillary (PTC) network, which causes the entire nephron to become hypoxic, thus triggering the expression of HIF‐1α. The OAT1 channel mainly exists on the tubular epithelial basement membrane of the kidney but is also present in small quantities in liver cells [28]. Our results showed that, following AA administration, XOR activation mainly occurred in the kidneys and was not observed in other tissues. Sun *et al*. [31] evaluated the PTC density as well as HIF‐1α and vascular endothelial growth factor (VEGF) expression in female rats that were administered dietary AA twice daily for eight weeks. They showed decreased PTC density over time, increased HIF‐1α and VEGF, and no improvement in VEGF expression. Sun *et al*. concluded that the AA‐mediated decrease in the PTC network resulted in higher HIF‐1α expression levels.

In the present study, AA administration significantly caused body weight loss, as well as renal function decline. A lower body mass index is associated with greater mortality in patients with CKD. Uremic cachexia is known to be the cause of weight loss in CKD, being associated with acidosis and inflammation. In addition, patients with CKD often have sarcopenia, which is related to increased risks of mortality and cardiovascular complications. Although we did not examine mouse food intake and skeletal muscle profiles in the present study, AA‐induced weight loss may have been caused by cachexia and sarcopenia. Cachexia caused by uremia was suggested to reduce food intake and body weight. On the other hand, the increase in adipogenesis and lipogenesis that occurred concomitantly with the increase in XOR activity, related to C/EBPα or PPARγ and SREBF1 gene expression progression, suggested that this reaction is a protective mechanism against weight loss.

We also observed that AA administration consistently increased the levels of HIF‐1α expression, likely due to tissue breakdown. Collectively, these results suggest that nephron hypoxia and increase in XOR may coincide. Persistently elevated expression levels of XOR in renal tissue were caused by AA‐induced apoptosis of the tubular epithelium. Tissue hypoxia led to the breakdown of ATP, thereby activating XOR. Elevated XOR activity was observed not only along with ATP breakdown, but during tissue damage caused by ROS production. Further investigation is required to determine whether XOR provoked tissue damage through production of oxidative stress. XOR is the rate‐limiting enzyme in purine metabolism. It produces uric acid and ROS, the latter of which generates oxidative stress. Through ROS production, pro‐inflammatory cytokines in the NLR family pyrin domain containing 3 (NLRP3) inflammasome/IL‐1β pathway are stimulated in macrophages [32]. Meanwhile, the NRLP3/IL‐1β pathway is also activated by monosodium urate, which exacerbates renal impairment through tissue deposition of uric acid crystals [32, 33, 34]. Yisireyili *et al*. [35] reported that in stress‐burdened C57BL/6 mice, XOR, MCP‐1, and TNF‐α expression levels were elevated in the visceral adipose tissue, with an increase in tissue F4/80 and CD68 staining, which could be reversed with febuxostat. Page *et al*. [36] evaluated the XOR activity induced by inflammatory cytokines in human mammary epithelial cells. XOR activity was mainly increased upon treatment with IFN‐γ, but no reaction was observed with IL‐6. Fibrosis is presumed to progress through enhanced stimulation of the TGF‐β pathway by transglutaminase type 2 (TG2), which promotes collagen I‐IV expression in the nuclear factor kappa‐light‐chain‐enhancer of activated B cells (NF‐κB) [37, 38, 39, 40]. When studying AA nephropathy in mice with a C57BL/6 background, Scarpellini *et al*. [41] reported that syndecan‐4, which promotes fibrosis, externalizes TG2 to the cell membrane. This results in the formation of a complex with TGF‐β and leads to the progression of kidney fibrosis. This effect is significantly attenuated in syndecan‐4 knockout mice. The proteasome inhibitor bortezomib was reported to inhibit fibrosis through TGF‐β inhibition [30]. TGF‐β plays an important role in fibroblast transformation in AA nephropathy through a fibrotic mechanism [4]. In our AA study, TGF‐β and XOR were expressed simultaneously, but this fibrotic mechanism was most likely induced through TGF‐β expression via activated XOR. The expression of TGF‐β and Col 1 genes was promoted in response to treatment with AA, which suggested a fibrotic mechanism of action. Moreover, the expression of HIF‐1a, a component of the hypoxia‐inducible factor, increased concurrently with XOR activation in the fibrotic pathway, which consequently increased oxidative stress in the tissues.

The findings of this experiment indicate that the production of reactive oxygen species (ROS) upon activation of the NADPH oxidase occurred concurrently with XOR activation in response to tissue necrosis caused by tissue hypoxia, followed by progression to the fibrotic pathway. Additionally, the microscopic observations showed that XOR activity and the fibrotic area were significantly correlated (Fig. 6C), suggesting that XOR might have caused partial tissue damage. However, further examination is needed.

It has been pointed out that XOR may regulate adipogenesis and mesenchymal transformation in the renal tubule epithelium [42]. However, it has not been clarified whether the XOR gene negatively regulates the adipose droplet deposition and mesenchymal transformation, or they result from xanthin deposition in kidney tissue when XOR is depleted. We did not investigate the serum lipid mechanism, but our study showed that the primary changes in renal tissue were apoptosis, a drop in tubular epithelial cells, and fibrosis. Lipid droplet deposition was not indicated. In this AA model, adipogenesis and lipogenesis increased concomitantly with XOR activity, contrarily to the XOR ‐/‐ model. This result suggested that the adipogenesis and lipogenesis that occur with C/EBPα or PPARγ and SREBF1 gene progression was the result of cachexia or inflammation caused by ROS, and not the work of XOR directly.

Chen et al. [43] reported that XOR activity was attenuated in C/EBP‐silent cells; therefore, C/EBP gene lies upstream of XOR gene. Additionally, in the ob/ob mouse model, XOR gene depletion was observed to inhibit adipogenesis; based on this, XOR was considered to lie upstream of the PPARγ gene. XOR is suggested as a therapeutic target of metabolic syndrome with hyper uricemia. In our AA model, PPARγ, C/EBP α, and SREBF1 gene expression were elevated concurrently with XOR activity. This result suggested that XOR activated adipogenesis or lipogenesis, or, conversely, that cachexia or oxidative stress caused by AA resulted in the activation of adipogenetic or lipogenetic genes as a protective mechanism; further investigation is required on this point. Administering AA caused apoptosis and nephron hypoxia with elevated HIF‐1α expression, and induced progression to the fibrotic pathway through TGF‐β and Col 1. XOR was induced mainly by hypoxia with oxidative stress, further suspected to produce ROS through elevated levels of NADPH oxidase, suggesting that XOR participated in the fibrotic mechanism. Further investigation on this mechanism is required to clarify whether adipogenesis and lipogenesis were induced by XOR or elevated as a consequence of tissue damage.

The main limitation of this study is the small group sizes. However, we observed clear differences in the markers examined, indicating that further investigation of therapeutic interventions is warranted. Further studies should evaluate the effect of XOR inhibition concerning the suppression of fibrosis in progressive renal diseases. Another limitation is that we did not investigate the serum lipid mechanism thoroughly and could not measure the serum lipid metabolic marker. However, the major changes observed in the renal tissues were apoptosis, a decline in the number of tubular epithelial cells, and fibrosis, while lipid droplet deposition was not shown. Further, we assessed adipogenesis and lipogenesis renal gene expression, which allowed us to evaluate lipid metabolism under AA administration. In this AA model, adipogenesis progressed concomitantly with increasing XOR activity, in contrast to what was observed in the XOR ‐/‐ model. This result suggested that XOR did not exert a direct protective function on renal tissues, and the fact that the adipogenesis that occurred with C/EBPα or PPARγ and SREBF1 gene expression progression resulted from inflammation induced by ROS suggested this adipogenetic or lipogenetic reaction is a protective mechanism.

In conclusion, the results of the present study showed that tissue XOR activity, caused by tissue hypoxia‐induced catabolism, was consistently activated in an AA‐induced nephropathy CKD model. Furthermore, kidney‐specific XOR activation was associated with organ damage and renal fibrotic lesion development. Thus, this AA nephropathy model could be useful to further investigate a preventive mechanism against the XOR‐mediated renal fibrotic changes leading to end‐stage renal disease.

## Conflicts of interest

The authors declare no conflict of interest.

## Author contributions

TI and TK performed the conceptualization, methodology, formal analysis and investigation, and wrote original draft. TI, TK, and HW reviewed and edited the manuscript; KT and HW involved in funding acquisition; TI performed the resources; SU, ST, EA, TS, TY, SK, RK, KH, TN, and SK supervised the manuscript.

## Data Availability

Data will be available from the corresponding author upon reasonable request.

## Data Accessibility

Data will be available from the corresponding author upon reasonable request.
